# The Effect of the Carbon Fiber Content on the Flexural Strength of Polymer Concrete Testing Samples and the Comparison of Polymer Concrete and U-Shaped Steel Profile Damping

**DOI:** 10.3390/ma12121917

**Published:** 2019-06-14

**Authors:** Ondrej Petruška, Jozef Zajac, Vieroslav Molnár, Gabriel Fedorko, Jozef Tkáč

**Affiliations:** 1 The Faculty of Manufacturing Technologies with the seat in Prešov, Technical University of Košice, Bayerova 1, 080 01 Prešov, Slovakia; ondrej.petruska@tuke.sk (O.P.); jozef.zajac@tuke.sk (J.Z.); jozef.tkac@tuke.sk (J.T.); 2 The Faculty of Mining, Ecology, Process Control and Geotechnologies, Technical University of Košice, Letná 9, 042 00 Košice, Slovakia; gabriel.fedorko@tuke.sk

**Keywords:** polymer concrete, flexural strength, damping

## Abstract

This article explores the effect of carbon fiber content on the flexural strength of polymer concrete testing samples and compares the damping of polymer concrete and U-shaped steel profiles. The experiments involved and described herein consisted of flexural strength testing according to STN EN 12 390-5 Testing of Hardened Concrete, Part 5: Flexural Strength of Test Samples. The test results were evaluated graphically and by calculations and were further processed in various programs. The experimental results indicated that the highest flexural strength value was obtained by the test samples containing 12% of carbon fibers while culminating at 17.9 MPa. The results showed that the highest increase of flexural strength was caused by the addition of 3% of carbon fibers to the mixture, which increased the flexural strength by 4.2 MPa, or 26.75%. The results indicated that, based on the shape of the regression curve, flexural strength culminated at 13% carbon fiber content. The experimental results demonstrated that the tested polymer concrete test sample had a 6.87 times higher attenuation coefficient than the U-shaped steel profile. The results showed that the polymer concrete test sample No. 4 reduced vibration acceleration deviation by 93.5% in 0.005 sec and the U-shaped steel profile by 32.9%.

## 1. Introduction

Increasing the performance of electric motors used to drive spindles and other moving machine parts for chip machining with a defined cutting edge, e.g., milling machines, lathes, and drills, also increases the rigidity of construction and vibration damping of these machines. Conventionally used materials, such as steel and cast iron, have enough rigidity, but vibration damping is very low. In the case of chip machining, vibrations arise mainly due to deceleration and acceleration of the worktable drive due to the rotational movement in the motor and the spindle as well as in the chip removal itself. As a result, vibrations spread in the machine frame, which adversely affects both the manufacturing process and the machine and its components. The solution that increases vibration damping is the replacement of conventionally used materials with a material with a higher attenuation coefficient. The material with good damping and strength is polymer concrete (PC).

Polymer concrete is a modern composite material that has been used in engineering since the 1970s. Polymer concrete is a mixture of filler, binder, and additives. The individual components are combined to achieve the desired properties for a specific application.

Many scientists around the world are exploring the properties of polymer concrete. In 2019, Mr. Ding and others provided a study, *Pressure Sensitivity of Smart Polymer Concrete Based on Steel Slag*. Their research provided us with a new type of smart polymer concrete block as a result of adding graphite and steel slag in the epoxy resin concrete to test the pressure-sensitive characteristics under uniaxial compression. The results showed that the resistance of the concrete with complex graphite and steel slag increased with the increase of strain under uniaxial compression, and the process showed concordant monotonicity [1,2]. A shape memory polymer concrete crack closure system activated by electrical current was investigated by Teallet et al. [3]. This stress was previously found to enhance the load recovery associated with autogenous self-healing. The above mentioned research provides the details of the experiments undertaken to incorporate SMP (shape memory polymers) structural joints containing polyethylene terephthalate (PET) filaments into reinforced and unreinforced 500 × 100 × 100 mm structural concrete beam samples. These structural joints are activated via an electrical supply using a nickel-chrome resistance wire heating system. Hassaniet et al. [4] investigated the effect of basalt, silica sand, and fly ash on the mechanical properties of quaternary polymer concretes. The aim of the Hassaniet study was to manufacture quaternary PCs and optimize the weight percentages of the epoxy resin, the ultrafine fly ash, the silica sand, and the basalt aggregates. They studied mechanical properties such as compressive, flexural, and splitting tensile strength. Chunwaet et al. [5] published the first ever experimental study on square multi-tube concrete columns. The test results demonstrated that the concrete in the square multi-tube concrete columns was very effectively confined by the multiple tubes, and that the buckling of the internal steel tubes was completely prevented, leading to full structural utilization of the materials and a very ductile response. Seenappaet et al. [6] dealt with gamma, X-ray, and neutron shielding properties of polymer concretes. The shielding properties among the studied different polymer concretes were compared. A detailed study showed that barium polymer concrete was a good absorber for X-ray, gamma, and neutron radiation. An experimental study on the mechanical and the thermal properties of basalt fiber and nanoclay reinforced polymer concrete was conducted by Niakiet et al. [7]. First, the effect of chopped basalt fiber on the compressive, the flexural, the splitting tensile, and the impact strengths as well as the effect of different temperatures (up to 250 °C) on the strength of fiber reinforced polymer concrete were investigated experimentally. Basalt fiber improved the mechanical properties and increased the thermal stability of PC.

Niaki et al. [8] dealt with the mechanical properties of epoxy/basalt polymer concrete. They investigated the effects of the amount of crushed basalt aggregates on the compressive strength, the flexural strength, and the splitting tensile strength of a polymer concrete obtained by epoxy resin. They found that increasing the amount of epoxy resin to the basalt aggregates to 25 wt% improved mechanical properties of the concrete. After determining the optimum weight percentage of basalt in epoxy, the mechanical properties of the optimized PC were experimentally investigated at three different temperatures: 50 °C, 75 °C, and 100 °C. They found that the larger aggregate size resulted in higher compressive strength and lower flexural and splitting tensile strength. Heidari-Raraniet et al. [9] investigated the representative volume element (RVE) concept based on micromechanics. The RVE was composed of silica aggregates and epoxy matrix. Comparison of numerical and experimental results showed that: (1) the more interfacial strength and fracture energy increased, the more the compressive strength of PC increased; (2) the compressive behavior of PC was highly dependent on the aggregate volume fraction and the distribution in comparison to aggregate shape, (3) the model had appropriate accuracy in predicting the compressive behavior of PC. Chandrika et al. [10] investigated Bremsstrahlung shielding parameters in polymer concretes. The detailed study indicated that the barium polymer concrete had a large bremsstrahlung dose rate and more specific bremsstrahlung constant values than the other studied polymer concretes. Yiyanet et al. [11] dealt with the study entitled Bond Behavior of Wet-Bonded Carbon Fiber-Reinforced Polymer-Concrete Interface Subjected to Moisture. This research assessed the effects of moisture (i) during carbon fiber-reinforced polymer (CFRP) application and (ii) throughout the service life. Before CFRP bonding, the concrete blocks were preconditioned with a water content of 4.73% (this is called “wet-bonding”). Three different epoxy resins were applied to study the bond performance of the CFRP–concrete interface when subjected to moisture (95% relative humidity). A total of 45 double-lap shear specimens were tested at the beginning of exposure and again after one, three, six, and 12 months. All specimens with normal epoxy resins exhibited adhesive failure. The failure mode of specimens with hydrophobic epoxy resin changed from cohesive failure to mixed cohesive/adhesive failure and to adhesive failure according to the duration of exposure. The study, Statistical Analysis of 3-Point Bending Properties of Polymer Concretes Made from Marble Powder Waste, Sand Grains, and Polyester Resin was investigated by Benzannacheet et al. [12]. The mechanical performance of concrete polymer beams subjected to three-point bending was investigated. The results obtained showed that the type of sand and the amount of marble powder and sand aggregate affected the resistance of the polymer concrete beams significantly. The marble waste increased their bending strength by reducing the porosity of polymer concrete.

Szajerskiet et al. [13] investigated the quantitative evaluation and leaching behavior of cobalt immobilized in sulfur polymer concrete composites based on lignite fly ash, slag, and phosphogypsum. Nine different sulfur polymer composites (SPC) containing radioactive Co-60 were prepared by hot mixing and pressing in order to determine the possibility of radioactive cobalt immobilization in the SPC matrix. Formulations of SPC were tested against Co-60 immobilization efficiency according to a slightly modified ANSI/ANS 16.1 leaching test (measurement of the leachability of solidified low-level radioactive wastes by a short-term test procedure). Results indicated very good immobilization efficiency for SL (lignite slag) and FA (fly ash) based SPC formulations and worse parameters regarding phosphogypsum based matrices. Kwon et al. [14] investigated polymer concrete periodic meta-structure to enhance damping for vibration reduction. This study presented a complex periodic structure composed of the cement concrete embedded with a periodic arrangement of polymer concrete. Experiments and vibration analysis were carried out to determine the dynamic properties and the flexural strength of the complex concrete according to embedment structure of polymer concrete. The study, Strength Developments and Deformation Characteristics of MMA-Modified Vinyl Ester Polymer Concrete was performed by Jinet et al. [15]. The modulus of elasticity tended to decrease as MMA (methyl methacrylate) contents increased, and curing temperatures decreased. Kim and Ibraheem [16] presented the efficacy of functional periodicity on controlling the occurrence of interfacial failure in concrete members strengthened with CFRP sheets. The hypothesis tested was that periodically placed stress reducers preserve the integrity of the CFRP–concrete interface by interrupting the progression of mechanical damage, unlike conventional debonding control methods based on a prescribed strain limit. Statistical inference alongside a probability-based assessment proved that the individual debonding control methods and their configurations affected the performance of the CFRP–concrete interface. Şimşek and Uygunoğlu [17] investigated a full factorial-based desirability function approach to the research of the optimal mixture ratio of polymer concrete. In this study, thermal, workability, and mechanical properties of polymers, such as thermoplastic polyurethane, polycarbonate, and polybutylene terephthalate mixed concrete, were analyzed and optimized with the use of full factorial design-based desirability function approach via Minitab((R)) version 15. The results showed that polycarbonate was the most attractive polymer to produce polymer concrete, which included low thermal conductivity. Hu et al. [18] dealt with enhanced flexural performance of epoxy polymer concrete (EPC) with short natural fibers. To enhance the flexural performance of EPC, two kinds of short natural fibers with high specific strength (sisal fibers and ramie fibers) were incorporated into EPC. The results of mechanical tests showed that a small content of natural fibers (0.36 vol%) could significantly increase the flexural strength of EPC by 25.3% (ramie fibers) or 10.4% (sisal fibers). Zegardlo et al. [19] produced the study, Physical and Mechanical Properties and Microstructure of Polymer Concrete with Recycled Glass Aggregate. Their research presented an analysis of the possibility of using glass waste from worn out lighting materials as an aggregate for polymer concrete. The results of the research showed that the aggregate obtained from glass waste could be successfully used for the production of a polymer concrete. The most beneficial physical and mechanical properties were obtained for a composite in which glass waste was used as a 50% substitute for traditional aggregate. In their study, Al Azzawi et al. [20] presented two full-scale concrete masonry walls that were repaired with three horizontally aligned 20 inch (508 mm) wide unidirectional carbon fiber sheets using different commercially available epoxies. Twenty years later, the carbon fiber-reinforced polymer concrete masonry unit (CFRP-CMU) bond was subjected to investigation through selective pull off tests that were preceded by detailed nondestructive evaluation. Results showed that, despite superficial damage to the top epoxy coating and debonding along masonry joints, the residual CFRP-CMU bond for the wall surface was largely unaffected by prolonged exposure to Florida’s harsh environment. Experimental studies of the manufacturing and the evaluation of mechanical, physical, and thermal properties of the polymer concrete mixtures were also addressed by other authors [21,22,23,24].

This study focuses on the effect of carbon fiber content on the flexural strength of polymer concrete test samples and on the comparison of the damping of polymer concrete and U-shaped steel profile. High flexural strength and attenuation coefficient together with high compressive strength are necessary material properties to use this composite material as a machine tool frame.

## 2. Materials and Methods

### 2.1. Materials

Eighteen polymer concrete test samples were manufactured for testing flexural strength. Three test samples of six types of compositions were manufactured according to [25]. This standard defines the shape, the dimensions, and other requirements for test samples and molds. To test flexural strength, test samples in the shape of a joist with dimensions of 100 × 100 × 500 mm were made. The test samples contained organic and inorganic fillers. Organic fillers were made by andesite gravel and silica sands of various fractions. Inorganic fillers consisted of carbon fibers in the form of dispersed reinforcement. Carbon fibers with the length of 3 mm were purchased from the Havel Composites CZ Ltd. Company. The characteristics of the organic fillers used are shown in Table 1.

The matrix was epoxy resin LH 160 and hardener H 287. Epoxy resin LH 160 is a low viscosity universal resin for room temperature processing and curing. It is also suitable for the production of fiber reinforced parts. Low viscosity improves moldability and allows use at low temperatures. Hardener H 287 provides a pot life of up to 5 h. The characteristics of these components are shown in Table 2.

The resin/hardener mixing ratio is 100/50 vol. The dosing accuracy must be within ±2 divisions. Adding a higher proportion of hardener does not produce faster or slower reactions but causes an insufficient cure that cannot be reversed in any way. The optimum processing temperature of the binder system is between 20–25 °C. Higher ambient temperature shortens pot life. An increase of 10 °C outside temperature shortens the pot life by half.

The U-shaped steel profile was chosen to compare the damping of the polymer concrete with conventional materials used for manufacturing of CNC (computer numeric control) machine frames. A U-shaped steel profile with the dimensions of 100 × 40 mm and a wall thickness of 6 mm was used.

### 2.2. The Ratio of the Components in the Mixtures

The filler was 75% and the binder was 25% of the volume of the mixture. The percentages of the filler are expressed in relation to volume. Weight-based dosing is mainly used for large filler fractions where a large portion of the container volume would be a gap between the stones. In this case, where the fractions from 0.06 to 8 mm were used, the gap was minimal. In epoxy resin LH 160 and hardener H 287 material sheets, the percentage was also based on volume. Therefore, volume-based dosing was chosen. Test samples No. 1–3 contained 50% andesite gravel, 30% silica sand ST 06/12, and 20% silica sand STJ 25 as fillers. It further contained epoxy resin LH 160 and hardener H 287 in a ratio of 100:50 as the binder. Test samples No. 4–6 had the same composition, but 3% carbon fibers were added and 1% of each organic filler was removed to maintain the filler to binder ratio. This was repeated for test samples No. 7–18, increasing the carbon fiber content to 15% for test samples No. 16–18.

### 2.3. Manufacturing Test Bodies

**Figure 1:**
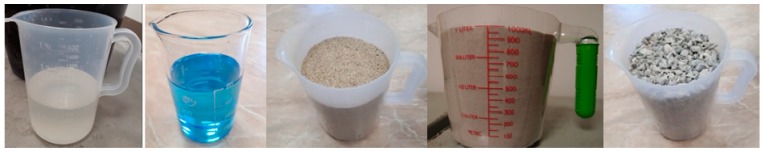
Used materials.

Test samples were manufactured at the Faculty of Manufacturing Technologies with the seat in Prešov. It was necessary to prepare molds before manufacturing. The inner walls of the cast iron molds were cleaned of impurities from previous production. Then, the inner walls of the molds were coated with Vaseline. This “separator” formed a non-stick layer on the inner walls of the mold, which ensured easy removal of the casting from the mold. Dosing was performed after preparation of the molds by measuring the volumes of used materials. Glass and plastic cups of suitable size were used for dosing, as illustrated in Figure 1.

**Figure 2:**
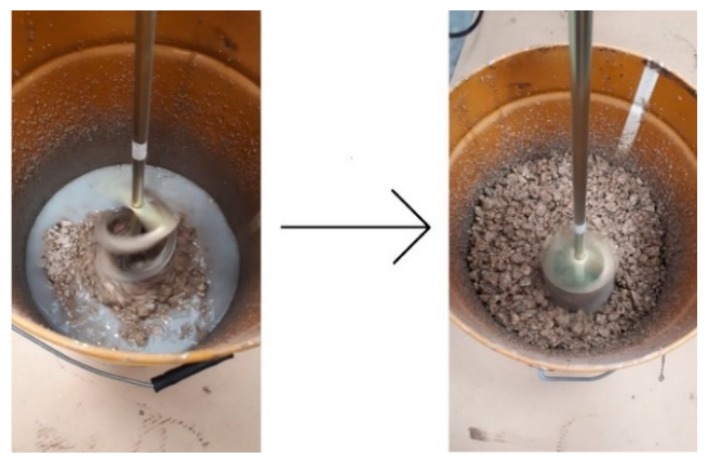
Matrix and fillers.

Mixing of the fillers and the binder was performed in two different containers. First, the fillers were mixed, which consisted of andesite gravel, silica sand, and carbon fibers. The mixing of the fillers ensured a uniform distribution of the individual kinds and fractions in the resulting mixture. Stirring of the binders (epoxy resin and hardener) was carried out for 3 min separately in a different vessel using a Makita UT1200 electric stirrer. The exothermic reaction started after the active hydrogen ions reacted with the epoxy groups. Then, the filler was gradually spilled under continuous stirring. The mixture of filler and binder was mixed until the matrix uniformly wrapped all the filler parts, as illustrated in Figure 2.

**Figure 3:**
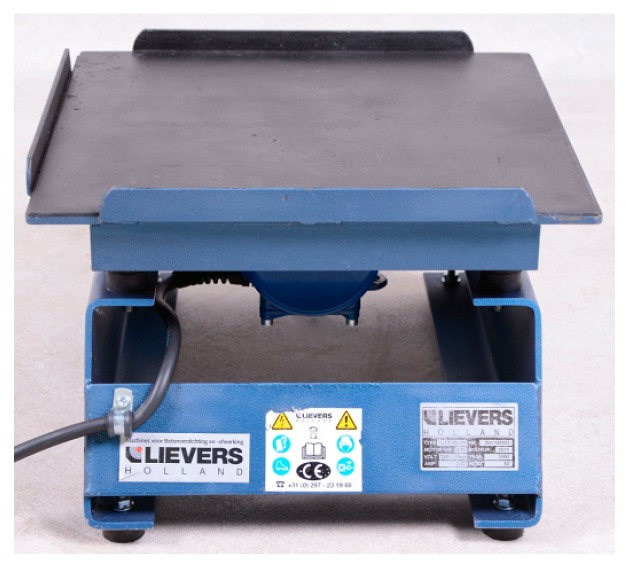
Table Lievers LTT 40/40.

After mixing, the mold was filled. The filled mold was vibrated on a Lievers LTT 40/40 vibrating table to compact the mixture and remove air bubbles. The Lievers LTT 40/40 table is shown in Figure 3.

**Figure 4:**
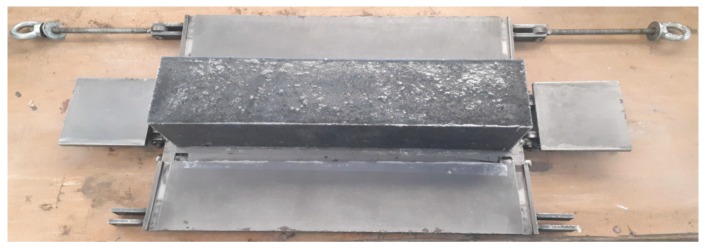
The casting from the mold.

After vibrating, the filled mold was placed on a horizontal surface. After 24 h, polymer concrete castings were removed from the molds, as illustrated in Figure 4.

For the next 10 days, the castings hardened. After this time, experiments were performed on the test samples.

### 2.4. Experimental Methods

**Figure 5:**
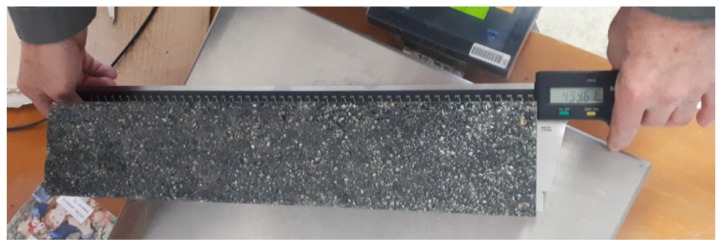
Control of test samples.

Eighteen test samples were tested by the Building Testing and Research Institute in the accredited testing laboratories in Prešov. The testing consisted of a flexural strength test according to the STN EN 12390-5 standard. In the beginning, the test samples were inspected according to the STN EN 12390-1 Testing of Hardened Concrete, Part 1: Shape, dimensions, and other requirements for test samples and molds. The dimensions of the test samples were controlled by a digital caliper with a large measuring range. The Mitutoyo ABSOLUTE AOS caliper, series 500 was used, as illustrated in Figure 5.

All test samples met the prescribed parameters. Subsequently, weighing of the test samples and determination of the specific weight was performed. Determination of the specific weight was carried out according to [27], Part 7: Specific weight of hardened concrete. This standard defines the requirements for the instruments used and the test procedure itself. It distinguishes three ways of setting the test specimen volume, two of which are:

- by immersion in water,
- by calculating from the actual measured dimensions.

**Figure 6:**
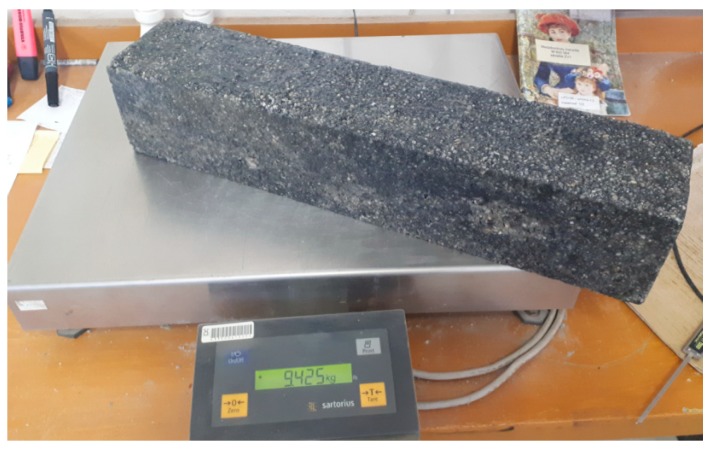
Weighing the test sample.

The method of determining the volume by calculating from the actual measured dimensions was chosen, since this measurement was already required in the previous step to check the shape and the dimensions of the test samples. The volume of test specimens was calculated from the actual measurements. Then, the test samples were weighed on a calibrated high-resolution digital weight. The Sartorius^®^-EA150-FEG-1 weight was used for this purpose. Figure 6 shows the weighing of the test sample.

Based on the calculated volume of the test sample and the measured weight, the specific weight was calculated based on the formula:

(1) $$D=\frac{m}{V}$$

where *D* (kg·m^−3^) is the specific weight of the test sample, *m* (kg) is the weight of the test sample, and *V* (m^3^) is the volume of the test sample. The specific weight was rounded to the nearest 10 kg·m^−3^ according to the above-mentioned standard. Table 3 shows the measured dimensions, the weight, and the standard deviation of the test samples and the average specific weight for the test samples with the same composition.

After that, the flexural strength test according to the STN EN 12390-5 Standard-Testing of Hardened Concrete, Part 5: Flexural strength of test samples, was performed. Testing was carried out on a CONTROLS, model: 50-C1201 / *.

**Figure 7:**
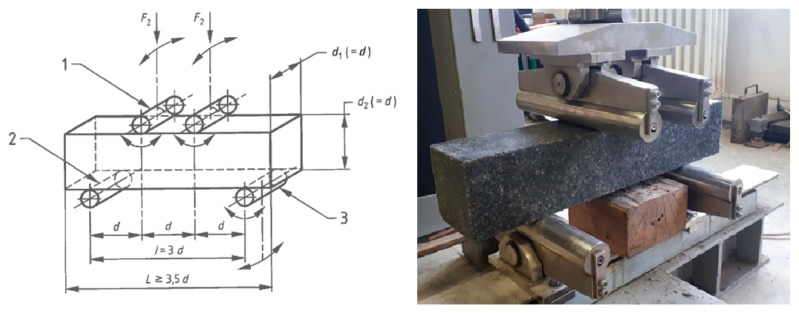
Test sample mounting scheme.

Before the test, it was necessary to adjust the distance of the rollers according to the diagram shown in Figure 6. The test sample thickness was 100 mm, thus the parameter d = 100 mm. The length of the test sample was 500 mm. Based on the diagram shown in Figure 7, the spacing of the upper pressure rollers was set up for 100 mm. The spacing of the lower support rollers was set up for 300 mm. The test sample was centered into the machine by the longitudinal axis perpendicular to the longitudinal axis of the rollers, as illustrated in Figure 7. The test sample was turned over the upper surface of the casting to the side. This provided a load direction perpendicular to the direction in which the polymer concrete was laid. The test sample loading rate was set to 0.05 MPa·s^−1^. The test sample was continuously loaded at the set speed until the maximum load was reached. The achievement of this limit was due to the transverse breaking of the test sample.

After a transverse break, the machine automatically recorded the maximum possible load and returned to its initial position. The flexural strength of the test samples was determined by the formula:

(2) $$\sigma_{FM}=\frac{M_{0,MAX}}{W_{0}},$$

where *σ_FM_* (MPa) is flexural strength, *M*_0,*MAX*_ (N.mm) is maximum bending moment, and *W*_0_ (mm^3^) is cross-sectional module. For a four-point bend, the maximum bending moment is defined by the formula:

(3) $$M_{0,MAX}=F_{MAX}\cdot L_{a},$$

where *M*_0,*MAX*_ (N.mm) is maximum bending moment, *F_MAX_* (N) is maximum force causing test sample transverse breaking, and *L_a_* (mm) is distance of the upper pressure rollers axes. The cross-sectional module is defined by the formula:

(4) $$W_{0}=\frac{b\cdot h^{2}}{6},$$

where *W*_0_ (mm^3^) is the cross-sectional module, *b* (mm) is width of the test sample, and *h* (mm) is thickness of the test sample. Flexural strength was given an accuracy of 0.1 MPa.

**Figure 8:**
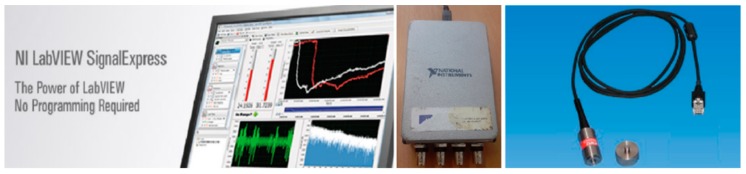
The NI LabVIEW SignalExppress software and the CMLV 3850 accelerometer.

Measurement of vibration damping was performed on test sample No. 2 before the flexural strength test. NI LabVIEW SignalExpress software from National Instruments and CMLV 3850 accelerometer were used for the measurement, as shown in Figure 8.

**Figure 9:**
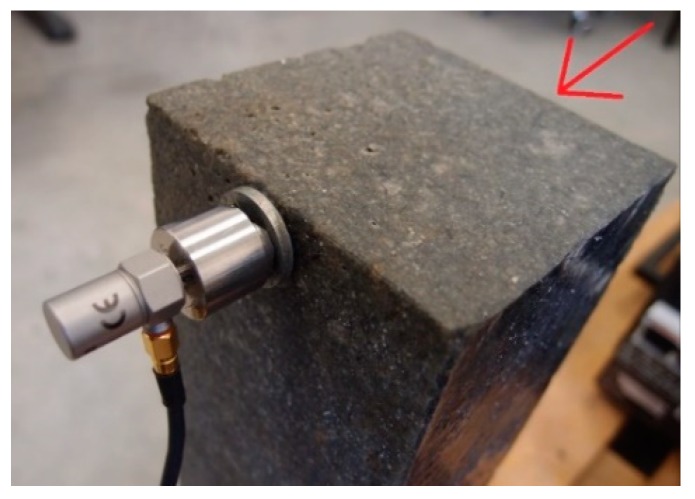
Location of the vibration acceleration sensor indicating the direction of stroke.

The CMSS 3811 sensor was glued to the top of the test sample. The hammer stroke on the opposite side of the casting caused oscillations that were recorded by the accelerometer, as shown in Figure 9.

**Figure 10:**
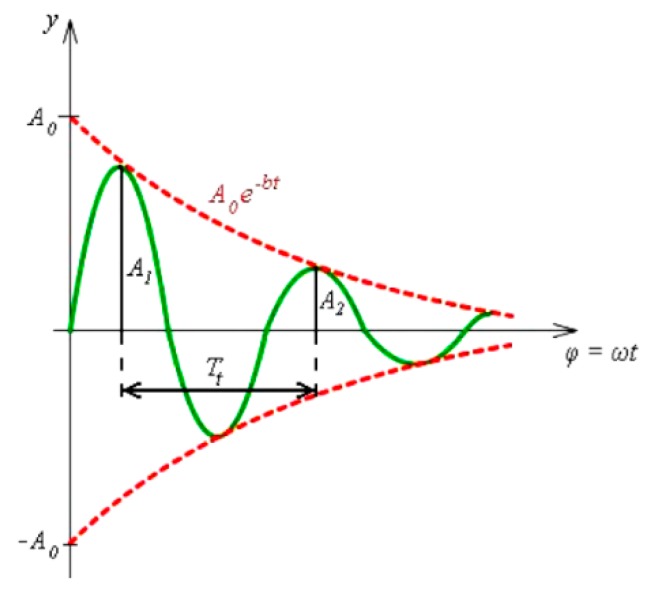
The schematic representation of attenuation λ in muted oscillation motion.

The solution of the motion equation of the attenuated oscillation was the equation describing the instantaneous displacement of the mass point from the equilibrium position, as shown by the formula [28]:

(5) $$A=A_{0}\cdot e^{-bT_{t}},$$

where *A* (m·s^−2^) is instantaneous displacement of the mass point, *A*_0_ (m·s^−2^) is initial amplitude, *e* is Euler’s number, *b* (-) is attenuation coefficient, and *T_t_* (s) is period. The λ attenuation is the ratio of two consecutive amplitudes of the same direction spaced by the period *T_t_*, as shown in Figure 10.

Then, the formula applies:

(6) $$\lambda =\frac{A_{2}}{A_{1}},$$

where *λ* (-) is attenuation, *A*_1_ (m·s^−2^) is highest amplitude, and *A*_2_ (m·s^−2^) is second highest amplitude of the same direction. The *λ* attenuation is a dimensionless number. The logarithmic decrement of attenuation *δ* is the natural logarithm *λ*. *δ* is a dimensionless number. The following formulas continue to apply:

(7) $$\delta =\ln\lambda =\ln e^{b\cdot T_{t}}=b\cdot T_{t}\cdot \ln e=b\cdot T_{t},$$

(8) $$\delta =b\cdot T_{t}\Leftrightarrow b=\frac{\delta }{T_{t}},$$

## 3. Results

Table 4 shows the measured maximum load force values, the standard deviation, and the average calculated flexural strength values for the test samples with the same composition.

**Figure 11:**
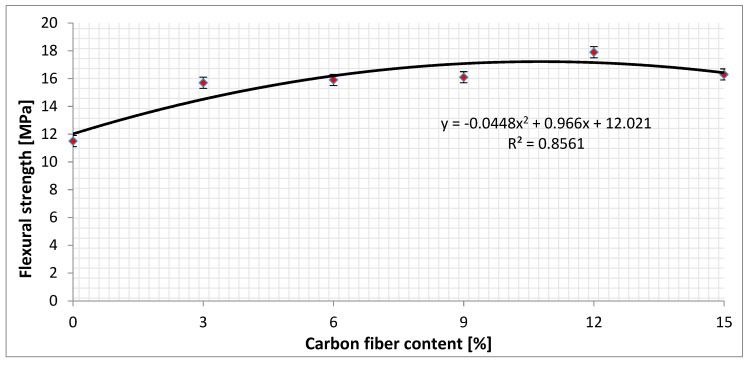
The graphical comparison of the effect of carbon fiber content on flexural strength.

Based on measured values of maximum load force and calculated flexural strength values, a graphical comparison of the effect of carbon fiber content on the flexural strength of polymer concrete test samples was created, as illustrated in Figure 11. An average of three test samples of the same compositions was used.

**Figure 12:**
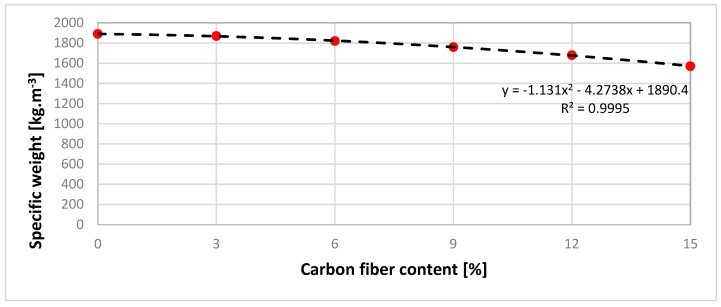
The graphical comparison of the effect of the carbon fiber content on specific weight.

Figure 11 shows the measured flexural strength values for six types of composition. The bars in Figure 11 show the precision of measured data on a CONTROLS, model: 50-C1201 / *. Precision of measured data was ± 0.3 MPa. The flexural strength as measured on test samples No. 1–3 without the carbon fibers reached a value of 11.5 MPa. The flexural strength as measured on test samples No. 4–6 with 3% of the carbon fibers reached a value of 15.7 MPa. This test sample experienced the highest increase of flexural strength by 4.2 MPa, or 26.75%. The test samples No. 7–9 containing 6% carbon fibers had a flexural strength of 15.9 MPa, and test samples No. 9–12 with a 9% carbon fiber had a flexural strength of 16.1 MPa. The highest flexural strength value was measured in 12% carbon fiber samples No. 13–15, which was 17.9 MPa. By further increasing the carbon fiber content to 15% for test samples No. 16–18, the flexural strength had already dropped to 16.3 MPa. Figure 11 also shows the regression curve represented by the second-degree polynomial function. The regression curve describes the measured points with an accuracy of 85.61%. To increase the accuracy of the regression curve, it is recommended to reduce the percentage difference in carbon fiber content between test sample compositions from 3% to 1.5%. Based on the shape of the regression curve, it could be stated that the flexural strength culminated at 13% carbon fiber content. Figure 12 shows the measured specific weight values for six types of composition. It also shows the regression curve represented by the second-degree polynomial function. The regression curve describes the measured points with an accuracy of 99.95%. Based on the shape of the regression curve, it could be stated that increasing the carbon fiber content reduced the specific weight of the test samples.

**Figure 13:**
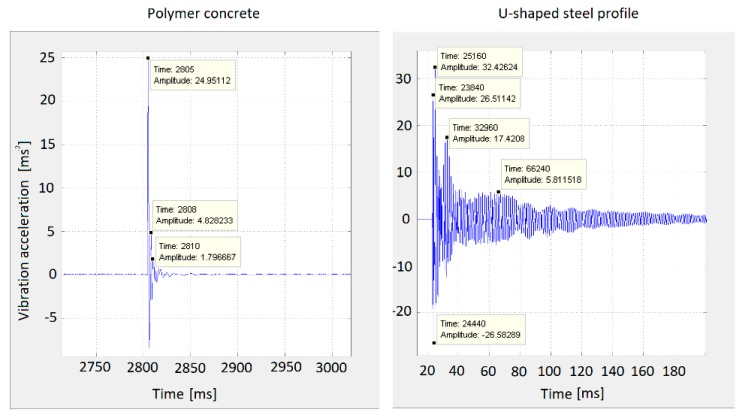
The comparison of damping in polymer concrete and U-shaped steel profile.

Figure 13 shows vibration acceleration measurement results. It contains a comparison of the damping properties of the two materials. The left part of the figure describes the damping of the polymer concrete test sample No. 4. The right part of the figure describes the damping of a U-shaped steel profile. From Figure 13, it can be seen that with almost the same deflection magnitude (vibration acceleration amplitude), the polymer concrete damping was many times higher than the steel damping. Based on the measured values shown in Figure 13, it was possible to substitute the values of the polymer concrete material to formulas (6)–(8).

$$\begin{array}{c} \lambda =\frac{A_{2}}{A_{1}}=\frac{4.828}{24.95112}=0.193498\\ \delta =\ln\lambda =\ln0.193498=-1.642864\\ b=\frac{\delta }{T_{t}}\frac{=-1.642864}{0.003}=547.495 \end{array}$$

It was also possible to substitute the U-shaped steel profile values.

$$\begin{array}{c} \lambda =\frac{A_{2}}{A_{1}}=\frac{17.4208}{32.42624}=0.537244\\ \delta =\ln\lambda =\ln0.537244=-0.6213\\ b=\frac{\delta }{T_{t}}\frac{=-0.6213}{0.0078}=79.654 \end{array}$$

The results showed that the attenuation coefficient *b* in the case of the steel profile was 79.654, and in the case of the polymer concrete test piece, it was 547.495. The results confirmed the excellent damping properties of the polymer concrete against steel. The composition of polymer concrete test sample No. 2 with 3% carbon fiber had a 6.87 times higher attenuation coefficient than the U-shaped steel profile.

In order to verify the calculated values of the attenuation of the polymer concrete and the steel profile, it was necessary to replace them in relation (5) and to derive the initial amplitude *A*_0_.

For the variable *A*, we substituted the measured values of the instantaneous displacement of the mass point over time, i.e., the measured values of the vibration acceleration *A*_1_ and *A*_2_, and derived the initial amplitude *A*_0_. Based on two measured vibration acceleration values at different times, the correctness of the calculation for polymer concrete was confirmed.

$$\begin{array}{c} A_{0}=\frac{A}{e^{-bT_{t}}}=\frac{24.95112}{e^{-547.3495.0,001}}=43.1384\;\left(m\cdot s^{-2}\right)\\ A_{0}=\frac{A}{e^{-bT_{t}}}=\frac{4.828233}{e^{-547.3495.0,004}}=43.1404\;\left(m\cdot s^{-2}\right) \end{array}$$

The results showed that the calculated initial amplitude *A*_0_ for the calculation of the amplitude *A*_1_ had a vibration acceleration value of 43.1384. The calculated initial amplitude *A*_0_ for the calculation of the amplitude *A*_2_ had a vibration acceleration value of 43.1404. The difference in the calculated initial amplitude value *A*_0_ was 0.002 m·s^−2^, or 0.0047%. The calculations showed that the measured values coincided with the calculated values and confirmed the accuracy of the calculated values of attenuation polymer concrete. Then, the steel profile results were confirmed.

$$\begin{array}{c} A_{0}=\frac{A}{e^{-bT_{t}}}=\frac{32.42624}{e^{-79.654.0,002}}=38.0259\;\left(m\cdot s^{-2}\right)\\ A_{0}=\frac{A}{e^{-bT_{t}}}=\frac{17.4208}{e^{-79.654.0,008}}=38.0258\;\left(m\cdot s^{-2}\right) \end{array}$$

The results showed that the calculated initial amplitude *A_0_* for the calculation of the amplitude *A_1_* had a vibration acceleration value of 38.0259. The calculated initial amplitude *A_0_* for the calculation of the amplitude *A_2_* had a vibration acceleration value of 38.0258. The difference in the calculated initial amplitude value *A*_0_ was 0.0001 m·s^−2^. The calculations showed that the measured values coincided with the calculated values and confirmed the accuracy of the calculated values of attenuation in steel profile.

**Figure 14:**
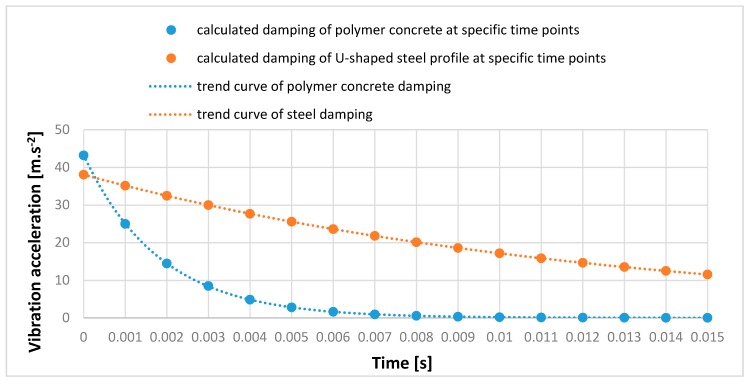
The comparison of calculated damping of polymer concrete and U-shaped steel profile.

A graphical comparison of the damping of the polymer concrete and the U-shaped steel profile was created based on their calculated damping values and amplitude height at specific time points, as shown Figure 14. Figure 14 confirms the excellent damping properties of the polymer concrete against the U-shaped steel profile as well as the results of the measured values shown in Figure 13. Polymer concrete test sample No. 4 reduced vibration acceleration deviation by 93.5% in 0.005 sec. After the same time, the U-shaped steel profile reduced vibration acceleration deviation by 32.9%.

**Figure 15:**
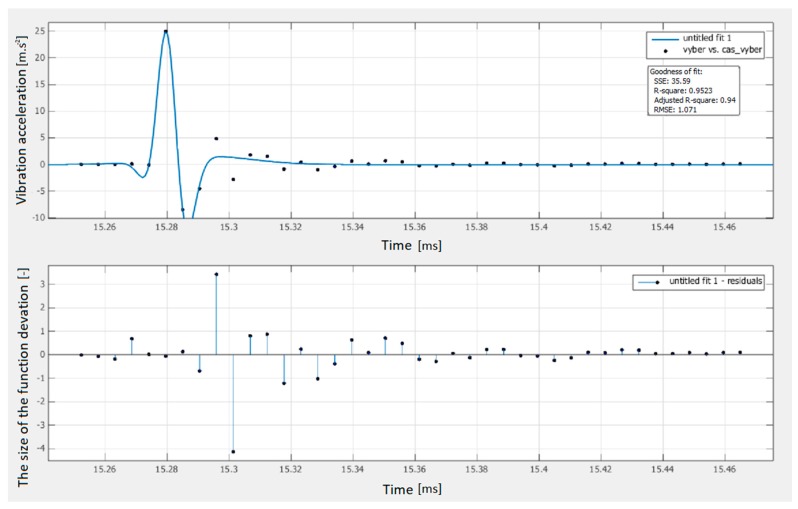
Gaussian regression curve.

The Gaussian regression curve was determined by Curve Fitting Tool software based on the measured values of the vibration acceleration of the polymer concrete test sample. The regression curve described the measured points with an accuracy of 95.23%, as shown in Figure 15.

The shape of the regression curve is defined by the function:

(9) $$f\left(x\right)=a_{1}\cdot \exp(-{\left(\frac{x-b_{1}}{c_{1}}\right)}^{2})+a_{2}\cdot \exp(-{\left(\frac{x-b_{2}}{c_{2}}\right)}^{2}+a_{3}\cdot \exp(-{\left(\frac{x-b_{3}}{c_{3}}\right)}^{2}$$

where the variables have the following values:

$$\begin{array}{ccc} a_{1}=6.533\times 10^{4} & b_{1}=-1.225 & c_{1}=0.08677\\ a_{2}=-6.533\times 10^{4} & b_{2}=-1.225 & c_{2}=0.08679\\ a_{3}=1.598 & b_{3}=-1.042 & c_{3}=0.3304 \end{array}$$

## 4. Conclusions

Polymer concrete is a modern composite material whose properties can be influenced by appropriate composition. This article focused on the effect of carbon fiber content on the flexural strength of polymer concrete test samples and the comparison of damping (in) a polymer concrete and a U-shaped steel profile. Adding carbon fibers as dispersed reinforcement to the polymer concrete test samples had a positive effect on flexural strength. Adding them to the polymer concrete test samples reduced the specific weight. The following are the key findings of the study:

- The highest increase of flexural strength was caused by the addition of 3% carbon fibers to the mixture, which increased the flexural strength by 4.2 MPa, or 26.75%.
- The highest flexural strength value was obtained by the test samples containing 12% carbon fibers while culminating at 17.9 MPa.
- Based on the shape of the regression curve, it could be stated that the flexural strength culminated at 13% carbon fiber content.
- Composition of polymer concrete test sample No. 4 with 3% carbon fiber had a 6.87 times higher attenuation coefficient than the U-shaped steel profile.
- Polymer concrete test sample No. 4 reduced vibration acceleration deviation by 93.5% in 0.005 sec. After the same time, the U-shaped steel profile reduced vibration acceleration deviation by 32.9%.

The composition of the investigated polymer concrete was designed to use this material for the manufacturing of frames for CNC milling machines. Since the frame is particularly stressed for flexural strength and vibrations from moving parts, these parameters were monitored on test samples. Future results that will be measured on a CNC milling machine made of polymer concrete will be published in other articles.

## Figures and Tables

**Table 1:** The characteristics of the organic fillers.

Kind of Filler	Size of the Fraction	Specific Gravity	Water Absorption	Mining Area
Andesite gravel	4–8 mm	2400 kg·m^−3^	0.5–2.5%	Fintice, Slovak Republic
Silica sand ST 06/12	0.63–1.2 mm	2500 kg·m^−3^	0.1–0.3%	Mladějov, Czech Republic
Silica sand STJ 25	0.06–0.31 mm	2700 kg·m^−3^	0.1–0.3%	Mladějov, Czech Republic

**Table 2:** The characteristics of epoxy resin LH 160 and hardener H 287 [26].

Kind of Binder	Property	Value of Property
epoxy resin LH 160	Density at 25 °C	1.13–1.17 g·cm^−3^
	Viscosity at 25 °C	700–900 mPa·s
	Epoxy mass equivalent	166–182 g·mol^−1^
	Epoxy index	0.55–0.60 mol.1000g^−1^
hardener H 287	Density at 25 °C	0.93–0.96 g·cm^−3^
	Viscosity at 25 °C	80–100 mPa·s
	Amine number	450–500

**Table 3:** Measured dimensions, weight, and average specific weight of the test samples.

No. of Test Sample	Measured Dimensions	Weight of Test Sample	Standard Deviation	Average Specific Weight
1	99.8 × 100.2 × 500.1 mm	9.355 kg	8.165 × 10^−3^ kg	1890 kg·m^−3^
2	99.6 × 100.5 × 500.3 mm	9.365 kg		
3	99.7 × 100.3 × 499.5 mm	9.345 kg		
4	98.5 × 100.4 × 499.7 mm	9.220 kg	12.247 × 10^−3^ kg	1870 kg·m^−3^
5	99.9 × 100.2 × 499.9 mm	9.235 kg		
6	99.6 × 100.1 × 499.6 mm	9.205 kg		
7	100.2 × 100.3 × 499.6 mm	9.130 kg	2.356 × 10^−3^ kg	1820 kg·m^−3^
8	100.1 × 100.2 × 499.8 mm	9.135 kg		
9	100.2 × 100.2 × 499.7 mm	9.135 kg		
10	101.1 × 100.6 × 499.6 mm	8.920 kg	8.165 × 10^−3^ kg	1 760 kg·m^−3^
11	99.8 × 100.2 × 499.8 mm	8.930 kg		
12	100.0 × 99.8 × 500.1 mm	8.910 kg		
13	101.1 × 100.6 × 499.6 mm	8.575 kg	10.261 × 10^−3^ kg	1680 kg·m^−3^
14	99.6 × 99.9 × 499.8 mm	8.560 kg		
15	100.2 × 100.6 × 500.4 mm	8.585 kg		
16	103.1 × 100.7 × 499.6 mm	8.125 kg	11.025 × 10^−3^ kg	1570 kg·m^−3^
17	99.8 × 100.3 × 499.8 mm	8.115 kg		
18	101.2 × 99.6 × 499.5 mm	8.140 kg		

**Table 4:** The measured load force values and the average calculated flexural strength values.

No. of Test Sample	Measured Load Force	Standard Deviation	Average Calculated Flexural Strength
1	38.24 kN	0.588 kN	11.5 MPa
2	39.02 kN		
3	37.58 kN		
4	51.85 kN	0.367 kN	15.7 MPa
5	51.40 kN		
6	52,30 kN		
7	52.50 kN	0.515 kN	15.9 MPa
8	53.16 kN		
9	51.90 kN		
10	54.73 kN	0.294 kN	16.0 MPa
11	55.07 kN		
12	55.45 kN		
13	61.45 kN	0.211 kN	17.9 MPa
14	61.40 kN		
15	60.98 kN		
16	56.61 kN	0.062 kN	16.3 MPa
17	56.47 kN		
18	56.59 kN		
